# The Incidence of QT Prolongation and Torsades des Pointes in Patients Receiving Droperidol in an Urban Emergency Department

**DOI:** 10.5811/westjem.2020.4.47036

**Published:** 2020-07-02

**Authors:** Jon B. Cole, Samantha C. Lee, Marc L. Martel, Stephen W. Smith, Michelle H. Biros, James R. Miner

**Affiliations:** * University of Minnesota Medical School, Department of Emergency Medicine, Minneapolis, Minnesota; † Minnesota Poison Control System, Minneapolis, Minnesota; ‡ Hennepin Healthcare, Department of Emergency Medicine, Minneapolis, Minnesota

## Abstract

**Introduction:**

Droperidol carries a boxed warning from the United States Food and Drug Administration for QT prolongation and torsades des pointes (TdP). After a six-year hiatus, droperidol again became widely available in the US in early 2019. With its return, clinicians must again make decisions regarding the boxed warning. Thus, the objective of this study was to report the incidence of QT prolongation or TdP in patients receiving droperidol in the ED.

**Methods:**

Patients receiving droperidol at an urban Level I trauma center from 1997–2001 were identified via electronic health record query. All patients were reviewed for cardiac arrest. We reviewed electrocardiogram (ECG) data for both critically-ill and noncritical patients and recorded Bazett’s corrected QT intervals (QTc). ECGs from critically-ill patients undergoing resuscitation were further risk-stratified using the QT nomogram.

**Results:**

Of noncritical patients, 15,374 received 18,020 doses of droperidol; 2,431 had an ECG. In patients with ECGs before and after droperidol, the mean QTc was 424.3 milliseconds (ms) (95% confidence interval [CI], 419.7–428.9) before and 427.6 ms (95% CI, 424.3–430.9), after droperidol (n = 170). Regarding critically-ill patients, 1,172 received droperidol and 396 had an ECG. In the critically-ill group with ECGs before and after droperidol mean QTc was 435.7 ms (95% CI, 426.7–444.7) before and 435.8 ms (95% CI, 427.5–444.1) after droperidol (n = 114). Of 337 ECGs suitable for plotting on the QT nomogram, 13 (3.8%) were above the “at-risk” line; 3/136 (2.2%; 95% CI, 0.05–6.3%) in the before group, and 10/202 (4.9%; 95% CI, 2.4%–8.9%) in the after group. A single case of TdP occurred in a patient with multiple risk factors that did not reoccur after a droperidol rechallenge. Thus, the incidence of TdP was 1/16,546 (0.006%; 95% CI, 0.00015 – 0.03367%).

**Conclusion:**

We found the incidence of QTc prolongation and TdP in ED patients receiving droperidol to be extremely rare. Our data suggest the FDA “black box warning” is overstated, and that close ECG monitoring is useful only in high-risk patients.

## INTRODUCTION

Droperidol is a butyrophenone first-generation antipsychotic approved by the United States Food and Drug Administration (FDA) for the treatment of postoperative nausea and vomiting (PONV). 1 Over the past 30 years it has also become a cornerstone therapy for nausea and vomiting, 2 headache, 3,4 and agitation 5–7 in the emergency department (ED). 8 On December 4, 2001, the FDA issued a boxed warning (commonly called a “black box warning”) for droperidol, noting an association with QT prolongation and torsades des pointes (TdP), that recommended electrocardiogram (ECG) monitoring before and continued for 2–3 hours after droperidol administration, and that if QT prolongation (> 440 milliseconds [ms] for men, 450 ms for women) was present, droperidol not be administered. Despite the fact that the boxed warning was based primarily on post-marketing surveillance data (49% of which came from outside the US, 9 including 83% of all the reported fatalities), 10 the use of droperidol in US EDs decreased substantially after the warning was issued. 11,12 Sales of droperidol fell 90% within one year of the release of the boxed warning. 10,13

As the use of droperidol declined sharply in the first decade of the 21st century, the drug also became scarce even for institutions that continued to use droperidol routinely despite the warning. Manufacturing delays and shortages of raw materials were reported by drug companies, and droperidol became effectively unavailable to most hospitals by 2013. 14 In the winter of 2019, droperidol again became widely available in North America as one US manufacturer resumed production. 1 Since the re-introduction of droperidol, many hospitals have been faced with the decision of whether or not to return droperidol to hospital formularies, and how to systematically integrate the FDA warning into practice. This current scenario is reminiscent of the months immediately following the release of the boxed warning, affecting a variety of medical specialties. 15–17

We previously studied the relationship between droperidol administration and QT prolongation in our ED; however, these data were published only in abstract form. 18–20 As droperidol has returned to the US market and clinicians again must make decisions about the risk of QT prolongation, our data are relevant once more. Thus, the objective of this study was to report the incidence of prolonged QT interval or TdP in patients who received droperidol in the ED.

## METHODS

### Study Design

This was a retrospective, observational cohort study of patients presenting to our ED from January 1, 1997–November 30, 2001, who received parenteral droperidol for any indication. A subanalysis of critically ill patients receiving droperidol was also conducted from January 1, 1997–December 31, 2001. Our institutional review board approved this study.

### Study Setting and Population

This study was conducted at an urban, Level I trauma center, safety-net hospital with approximately 100,000 ED visits per year. Our patient population includes a large number of patients with substance use disorders; in fact, we have an entire unit within the ED to care for this patient population. 21 These patients are potentially high risk for either drug-drug interactions (such as cardiotoxicity with cocaine), or drug-disease interactions (such as an increased risk of hypokalemia in patients with alcohol use disorder that may predispose a patient to a prolonged QT interval that may be synergistic with droperidol), making our study population relatively high risk compared to other patient populations in which droperidol has been studied, such as those with PONV. During the study period approximately 2,500 patients per year received droperidol. 22

**Population Health Research Capsule**

What do we already know about this issue?

*Droperidol is again widely available, but still carries a Food and Drug Administration warning for QT prolongation and torsades des pointes (TdP) with restrictive monitoring and dosing recommendations*.

What was the research question?

What is the incidence of clinically meaningful QT prolongation and TdP in ED patients receiving droperidol?

What was the major finding of the study?

*QT prolongation was uncommon in ED patients receiving droperidol, and only 1 of 16,546 patients (0.006%) had TdP*.

How does this improve population health?

*The FDA warning is likely over-cautious; cardiac monitoring resources are probably best used only on patients at high risk for TdP*.

The most common indications for droperidol during this period in our ED from most to least common were acute agitation secondary to ethanol intoxication, non-headache pain, vomiting, and headaches. 3,22–24 Our ED includes a geographically separate critical care unit (CCU) that easily allows for identification of critically ill patients in a retrospective fashion. Determination of critical illness and placement of patients in the geographically separate CCU was at the discretion of the treating emergency physician. Our critical care rooms are never used for non-critically ill patients, making determination of critical illness by geographic location in a retrospective fashion relatively accurate. During the study period, our ED electronic health record (EHR) was EmSTAT (A^4^ Health Systems).

### Selection of Participants

We analyzed patients in two separate cohorts: those deemed critically ill, and those deemed not critically ill. Critically ill patients were defined as having undergone resuscitation in our previously described, designated CCU. 21 The location designation in the EHR made it simple to retrospectively determine who was considered critically ill at the time of their ED presentation. EmSTAT was queried for all patients who received droperidol in ED non-critical care areas from January 1, 1997–November 30, 2001. We then identified those who had an electrocardiogram (ECG) ordered after administration of droperidol. ECGs were reviewed and the computerized Bazett’s corrected QT intervals (QTc) were recorded. We analyzed ECG data for both critically ill and noncritical patients in three groups – patients with an ECG only before droperidol; only after droperidol; and those with ECGs both before and after droperidol.

To further analyze critically ill patients receiving droperidol, in addition to EmSTAT queries the medical records of all critically ill ED patients from January 1, 1997–December 31, 2001 were hand-searched for any patient receiving droperidol who also had an ECG performed during that visit. For critically ill patients, ECGs with bundle branch blocks or paced rhythms were excluded. We analyzed data in three groups: patients with an ECG only before droperidol; only after droperidol; and those with ECGs both before and after droperidol. We included in the analysis any ECG obtained in the ED after the administration of droperidol, regardless of its proximity to the administration of droperidol.

All subjects receiving droperidol were evaluated for the presence of any ventricular dysrhythmias, with the exception of premature ventricular dysrhythmias. Ventricular arrhythmias were identified via review of ECG interpretations that were recorded for usual care, as well as a query of the EHR for the diagnoses of TdP, ventricular fibrillation, or ventricular tachycardia.

### Outcome Measures

As a medication-induced, Bazett corrected QT of < 480 ms is generally considered safe, 25 we defined long QT by a Bazett corrected QT ≥ 480 ms. 26,27 Medical records of patients with long QT were further reviewed for previous ECGs demonstrating long QT or TdP. Cardiac monitoring and rhythm strips were reviewed during the course of usual care and may have contributed to final diagnoses, but were not specifically reviewed for the purpose of this study.

For critically ill patients, droperidol dose, ECG timing and intervals, and cardiac rhythms were recorded. Heart rates and corresponding raw QT intervals for patients with a heart rate <150 beats per minute (bpm) were measured manually and plotted on the QT nomogram 28 to assess the risk of drug-induced TdP. 29 The QT nomogram is a tool developed in the early 21st century that has superior sensitivity and specificity to most commonly used QTc cutoff numbers (including 500 ms). 29,30 As the vast majority of drug-induced TdP cases occur at heart rates between 30–90 bpm, 29 we also sought to report the number of patients who were “at-risk” on the nomogram in this heart rate range.

### Data Analysis

We analyzed data with descriptive statistics, chi-squared test and Fisher’s exact test, where appropriate.

## RESULTS

**Figure 1:**
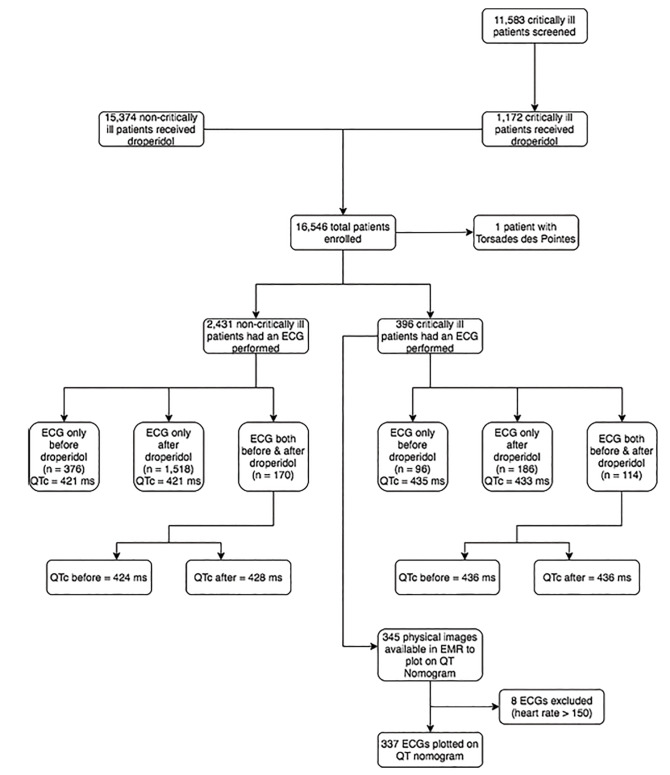
Study enrollment. QTc values, in milliseconds, in each box represent a mean value. *ECG*, electrocardiogram; *EMR*, electronic medical record; *QTc*, corrected QT interval; *ms*, millisecond.

Complete study enrollment is displayed in Figure 1. Of non-critical patients, 15,374 received 18,020 doses of droperidol in the ED; 2,431 of these patients also had an ECG. Of the patients with ECGs, 376 had an ECG before droperidol, 1,518 had an ECG after droperidol, and 170 had an ECG before and after droperidol. The mean QTc in patients with an ECG before droperidol treatment was 421.3 ms (95% confidence interval [CI], 418.0 – 424.6). The mean QTc in patients with an ECG after droperidol was 421.0 ms (95% CI, 419.5 – 422.5). In the group with ECGs before and after droperidol treatment, the mean QTc was 424.3 ms (95% CI, 419.7 – 428.9) and 427.6 ms (95% CI, 424.3 – 430.9), respectively. The mean ratio of the QTc before to after droperidol treatment was 1.009 (95% CI, 0.99 – 1.02).

**Figure 2:**
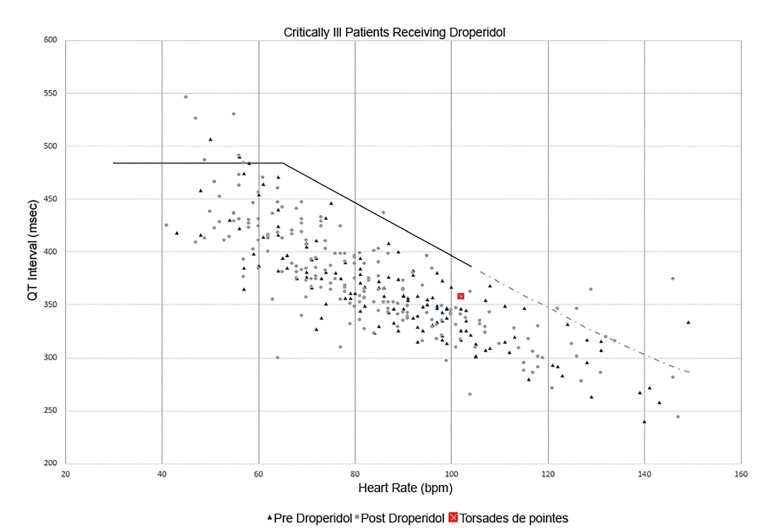
Heart rate and QT interval plotted on the QT nomogram. Data points above the line are considered “at-risk” for drug-induced torsades des pointes (TdP). Triangle represent electrocardiograms (ECG) obtained before patients received droperidol. Circles represent ECGs obtained after patients received droperidol. The lone square is the patient who experienced TdP; however, this ECG was obtained after defibrillation occurred and magnesium was administered. *ECG*, electrocardiogram; *bpm*, beats per minute; *msec*, millisecond.

Regarding critically ill patients, 11,583 charts were reviewed. Of these, 1,172 patients received droperidol and 396 had an ECG performed that did not have a bundle branch block or paced rhythm. In 96 patients an ECG was obtained only before droperidol; mean QTc was 435 ms (95% CI, 428.1–441.9 ms). In 186 patients an ECG was obtained only after droperidol; mean QTc was 433 ms (95% CI, 427.8 to 438.8 ms). In 114 patients ECGs were obtained before and after droperidol; mean QTc was 435.7 ms (95% CI, 426.7–444.7 ms) before droperidol and 435.8 ms (95% CI, 427.5–444.1ms) after droperidol. The mean ratio of the QTc before and after droperidol was 1.005 (95% CI, 0.985–1.025). Droperidol dosing data are displayed in the Table. Of the 396 critically ill patients who had ECGs performed, 345 physical images of ECGs were saved in EmSTAT that could be measured for the heart rate and RR interval. Of these 345, 7/138 (5.1%; 95% CI, 2.1 – 10.2%) had a QTc > 480 ms before droperidol, and 8/207 (3.9%; 95% CI, 1.7 – 7.5%) had a QTc > 480 ms after droperidol. Of 345 ECGs 8 were excluded for rates > 150 bpm, leaving 337 ECGs to plot on the nomogram (Figure 2). Of these, 13 patients (3.8%) were above the “at-risk” line; 3/136 (2.2%; 95% CI, 0.05 – 6.3%) in the before group and 10/202 (4.9%; 95% CI, 2.4% – 8.9%) in the after group. Eight patients (2.4%; 95% CI, 1.0 – 4.6%) with a pulse <90 bpm were above the “at-risk” line: two in the before-droperidol group and six in the after-droperidol group.

One patient of the 16,546 patients enrolled suffered cardiac arrest, deemed unrelated to droperidol. This patient, previously reported, 22 had a seizure followed by a cardiac arrest 11 hours after a single dose of droperidol in the ED. This patient had “stuffed,” or hastily ingested, an unknown amount of cocaine in an attempt to avoid being jailed and presented with agitation, which was treated with droperidol and lorazepam. The patient was resuscitated and discharged neurologically intact one week later. Given that the half-life of droperidol is 2.3 hours 31 and the clinical picture was consistent with cocaine toxicity, the treating team and the investigators deemed this cardiac arrest unrelated to droperidol.

Of the remaining patients, five experienced ventricular dysrhythmias, four had bigeminy, and one had TdP. The single case of TdP occurred in a patient with an alcohol use disorder who presented for nausea and vomiting; symptomatic TdP was observed on cardiac monitoring. The patient was then moved to a critical care room and defibrillated successfully after one shock; intravenous (IV) magnesium was administered. QTc post-defibrillation was 466 ms, and a post-defibrillation ECG was low risk when plotted on the QT nomogram. This patient was found to have hypomagnesemia and subsequently underwent electrophysiology testing including provocation with droperidol, which elicited QTc prolongation but no dysrhythmias. Thus, we found the incidence of TdP in ED patients receiving droperidol to be 1/16,546 (0.006%; 95% CI, 0.00015 – 0.03367%).

## DISCUSSION

In this cohort of 16,546 ED patients we found QT prolongation to be extremely rare. We found no clinically significant difference in QT interval among non-critically ill patients who had an ECG performed either before or after droperidol administration. Of a higher risk, critically ill cohort, we found the proportion of patients experiencing a QTc > 480 ms to be similar in patients before they received droperidol (5%) as in patients who had an ECG performed after droperidol (3.9%). When critically ill patients receiving droperidol had their ECGs plotted on the QT nomogram to stratify the risk of TdP, only 3.8% were deemed “at risk” for TdP. We observed a single case of TdP in a high-risk patient that was recognized and corrected before cardiac arrest occurred. Once stabilized, this patient did not have recurrent dysrhythmias after re-exposure to droperidol. Our data suggest that TdP with droperidol is extremely rare, and that when it occurs it does so in patients with multiple risk factors, such as a patient with an alcohol use disorder with an electrolyte disturbance who is actively vomiting, likely triggering a vagal bradycardic response.

Our data contribute to the existing data suggesting the risk of droperidol-induced dysrhythmias is exceedingly rare. Even at the time the FDA boxed warning was issued, peer-reviewed data did not support a solid link between droperidol and TdP, as demonstrated by one review that noted in 67,000 prescriptions for droperidol, not a single cardiac arrest was found. 32 In fact, at the time the FDA boxed warning was issued, the available peer-reviewed, indexed literature demonstrating any evidence regarding an association between droperidol and QT prolongation or TdP was composed of three clinical studies and seven case reports. 33 The FDA specifically cited two of these studies in their decision to add a boxed warning, both of which used larger doses than typically used in EDs. 34–36 One study randomized 40 head and neck surgical patients to three doses of IV droperidol (0.1, 0.175, and 0.25 milligrams per kilogram [mg/kg]) and observed a dose-dependent increase in the QT interval over a 10-minute study period. 36 The other study presented a case report of a patient who suffered TdP after 12.5 mg of IV droperidol, which occurred again after a droperidol re-challenge. The authors then went on to present a prospective observational study of 55 volunteers who received 0.25 mg/kg of IV droperidol prior to elective surgery and noted an increase from baseline in the QT interval in 70% of patients. 35 The sentinel patient experiencing TdP, however, later was determined to have bifascicular block needing a pacemaker, and the authors concluded their data was no reason to avoid the use of droperidol.

In addition to these two studies, the FDA also considered approximately 270 post-marketing surveillance reports submitted to MedWatch, the FDA’s Safety Information and Adverse Event Reporting Program. Multiple research groups subsequently submitted Freedom of Information Act requests to obtain and analyze these reports, 9,10,13,15,33,37 each of which helps clarify unique aspects of these cases. These analyses demonstrate clearly the MedWatch cases used to support the FDA boxed warning do not reflect the use of droperidol in a typical North American ED, nor are the reports of high quality. Several of these cases are duplicate reports (one cardiac arrest case was submitted five different times). 9 Accounting for duplication there are 232 unique cases. 10 Not all of these cases involved bad outcomes; of 273 reports, 127 involved a serious adverse event (SAE) (death, prolonged hospitalization, or a life-threatening condition) 37 including 94 deaths, 33 65 of which were associated with a cardiac sign or symptom. 9 Furthermore, not all SAEs were cardiac; of all reports, 97 involved a cardiac symptom, 9 including 11 patients with TdP (six of whom survived). 9,15

In addition, these cases do not reflect the use of droperidol in the US. Of these 127 SAEs, 74% came from sources outside the US, 37 including 83% of all the fatality reports; only 15 deaths came from within the US. 10 Dosing was also atypically large. Of the foreign-reported deaths that included dosing, 49% of them involved doses ≥50 mg, with some patients receiving up to 250 mg. 10 In total, only 14 deaths were reported at doses ≤5 mg. 33 Confounding concomitant medications or medical conditions were also extremely common; of the 14 deaths at doses ≤5 mg, in only two was droperidol the only medication given, 38 and in both cases either an alternative explanation was as likely or droperidol as a cause was not pharmacokinetically plausible. 33 One group conducted an in-depth analysis of all 10 reported deaths at doses ≤1.25 mg (only two of which were ED patients) and found that in none of the cases was a cause-and-effect relationship present. 13 Another used the Naranjo algorithm for adverse drug events to assign causality to all 65 cardiac deaths, and found no case scored higher than “possible cause” on the algorithm. 9 Last, the manner in which these cases were submitted to MedWatch was atypical. Of the approximately 270 reports, 71 were submitted on a single day (July 9, 2001), including 53 of the 94 deaths. 33 This large, single-day MedWatch submission came from Janssen-Cilag, 38 the company that until March 31, 2001, sold and marketed droperidol in Europe (but not the US). 15 Notably the mean interval from event to report of these cases submitted by Janssen-Cilag was 7.4 years, compared to 1.6 years for the remainder of the reports. 10

Since the issue of the black box warning, several studies have attempted to better quantify the risk of QT prolongation and TdP with droperidol. In a large anesthesia practice, the first-line drug for PONV changed from droperidol (before the boxed warning) to 5HT_3_ antagonists (eg, ondansetron) after the boxed warning. They found that out of 291,188 patients (16,791 of whom the authors estimated received droperidol, all in the “before” group), there were three unexplained deaths within 48 hours; one in the before (droperidol) group; and two in the after (mostly ondansetron) group. 39 In the single, unexpected fatality case where droperidol was used the patient died over 11 hours after a 1.25 mg dose of droperidol, making it extremely unlikely droperidol was responsible given its 2.3-hour half life. The same group later analyzed another 20,122 surgical patients who received 35,536 doses of droperidol and found no patients developed polymorphic ventricular tachycardia or death due to droperidol. 40

An Australian group prospectively evaluated 1,403 patients receiving ≥10 mg for acute behavioral disturbances in six different EDs, 1,009 of whom had ECGs within two hours of droperidol and found that only 13 patients were “at-risk” on the QT nomogram (seven of whom had other explanations for a long QT); no patients suffered a ventricular dysrhythmia or died. 41 Recently another American group evaluated 6,353 ED encounters where patients received droperidol and found the incidence of a QTc>500 ms was 1.2% in the six months prior to receiving droperidol, and 0.7% after receiving droperidol. 42 None of these patients suffered TdP or died. We recently published a review that included 4,947 patients who received a median dose of 5 mg intramuscular (IM) droperidol for acute behavioral disturbance from 2012–2013; no patients suffered cardiac arrest. 43

Our data align with the findings published since the boxed warning, that the incidence of clinically meaningful adverse cardiac events with droperidol is extremely rare, even in a critically ill, high-risk ED population. Although our data are not recent, they are again relevant as hospitals consider usage restrictions on droperidol now that it is widely available for the first time since 2013. Locally we have noted a substantial variation from institution to institution in terms of restriction of use and required monitoring. Some have not restricted the use of droperidol in any fashion, while some have instituted restrictions even more stringent than the boxed warning, including no use of “as needed” dosing, disallowing the use of droperidol on any order sets in the EHR, and application of the boxed warning’s ECG monitoring parameters for doses less than the FDA-approved 2.5 mg. (The FDA has since clarified that the boxed warning does not apply to doses less than the approved dose.) 44

Our own institution’s response has been to resume clinical use of droperidol as we once did, with no additional mandatory monitoring requirements. Interestingly, during a recent pharmacy residency accreditation visit from the American Society of Health System pharmacists, our institution was cited for not having measures in place to assure appropriate monitoring of medications with boxed warnings; droperidol was cited as a specific example. In response we have added language from the boxed warning into the medication order itself; however, as peer-reviewed published data do not support routine ECG monitoring this has remained a suggested (but not mandatory) practice. Of note, since the re-introduction of droperidol at our hospital in March 2019, we have administered 3,994 doses of droperidol and have had no adverse events reported to our medication safety committee.

## LIMITATIONS

This study has several limitations, including the usual limitations of a retrospective chart review, including convenience sampling and the possibility of unmeasured bias. An example of such a limitation is that because QT monitoring, such as with serial rhythm strips, was not done prospectively, it is possible that events of QT prolongation or even dysrhythmias were missed. Furthermore, we were unable to assess the relative frequency of complications compared to other therapies commonly substituted for droperidol in its absence 2,14,45,46 because of lack of a comparative group.

The age of our data itself may be a limitation. Although the objective nature of ECG intervals is unlikely to change over time, we have not used the EHR that contained these data since 2007. Many of the patient records in our study are no longer available for review, which limits our ability to conduct additional analysis. The QT nomogram had not been invented at the time our data were collected. 29 Once plotted on the nomogram our data suggested 3.8% of critically ill patients receiving droperidol were above the “at-risk” line for TdP. Because the records of these patients are no longer available, we are unable to further analyze these 13 patients to determine whether they had additional risk factors for QT prolongation. Alternatively, the age of our data may carry a unique advantage. All of the patients in the present study received droperidol before the publication of the boxed warning, and as such represent a unique cohort not subject to selection bias that may have pushed emergency physicians to avoid droperidol in at-risk patients, such as those with electrolyte disturbances or underlying cardiac disease. Such bias could make droperidol appear safer than it actually is. A cohort of ED patients receiving droperidol from 1997–2001 may represent a higher risk group than would be seen in a present-day study, and as such may allow for a “worst case” estimate of the incidence of torsades des pointes.

An additional limitation is the use of Bazett’s QT correction. Because the risk of drug-induced TdP is directly proportional to the heart rate (bradycardia prolongs the vulnerable period where a depolarization could trigger TdP) Bazett’s correction over-estimates the risk of the QT interval in tachycardic patients, and under-estimates the risk in bradycardic patients. 26 Nevertheless, Bazett’s correction is commonly used in the droperidol literature, 42 and is the most common formula used by toxicologists to risk stratify patients for TdP. 47 We attempted to account for this limitation by using the QT nomogram, a tool with greater sensitivity and specificity for detecting drug-induced TdP. 29 Last, we studied primarily patients receiving droperidol doses in the 2.5 – 5 mg range. We therefore cannot make generalizations about larger droperidol doses. Calver et al, however, used 10 mg of IM droperidol in a high-risk ED population with acute behavioral disturbance and found no cases of TdP and only six patients above the “at-risk” line on the QT nomogram. Subsequent studies from Australia have found these larger doses to also be safe. 48,49

## CONCLUSION

We found the incidence of QTc prolongation and torsades des pointes in ED patients receiving droperidol to be extremely rare. The sole case of TdP we found had multiple risk factors for dysrhythmias. Our data suggest the FDA black box warning is overstated, and that close monitoring of patients is useful only in high-risk patients, such as those with critical illness and multiple risk factors for TdP.

## Figures and Tables

**Table:** Characteristics and electrocardiogram data for critically ill patients receiving droperidol (n = 396).

	ECG before Droperidol (n = 96)	ECG after Droperidol (n = 186)	ECG before and after Droperidol (n = 114)
Median QTc (Bazett’s correction)	424 ms (range, 353 – 526)	424 ms (range, 309 – 533)	Before: 428 ms (range, 353 – 526) After: 423 ms (range, 309 – 533)
Mean time to ECG	33.3 minutes before	25.9 minutes after	Before: 28.2 minutes After: 108.8 minutes
Mean droperidol dose	2.75 mg	3.68 mg	2.21 mg
Ventricular dysrhythmias			
Bigeminy	2	2	-
Torsades de Pointes	-	1	-

*ECG*, electrocardiogram; *QTc*, corrected QT intervals; *ms*, milliseconds; *mg*, milligrams.
